# A qualitative evaluation of older people’s perceptions towards optimal diet management in the context of antimicrobial resistance

**DOI:** 10.1186/s12889-025-25375-4

**Published:** 2025-11-19

**Authors:** Lorna Flintham, Charlotte Dack, Francoise Koumanov, Edward Feil, Ben Ainsworth

**Affiliations:** 1 Department for Health, Faculty of Humanities and Social Sciences, University of Bath, Bath, UK; 2 Department of Psychology, Faculty of Humanities and Social Sciences, University of Bath, Bath, UK; 3 Department of Life Sciences, Faculty of Science, University of Bath, Bath, UK; 4 Department of Psychology, Faculty of Environmental and Life Sciences, University of Southampton, Southampton, UK

**Keywords:** Antimicrobial resistance, Health behaviour, One health, Community engagement, Motivation, Older adults, Gut microbiome

## Abstract

**Introduction:**

Antimicrobial Resistance (AMR) poses a significant global public health problem. The risk of developing a reservoir of antimicrobial resistant genes in the gut is reportedly higher with certain diets, rendering individual diet-related AMR management a largely untapped approach in AMR mitigation. As a vulnerable population, older people are at particular risk of poorer resistant infection outcomes. The aim of this study was to explore older people’s perceptions of diet and health factors surrounding AMR.

**Method:**

This was a qualitative study using semi-structured interviews. Purposive convenience sampling was used to recruit 17 members of the public who were aged 65 years and over and a basic questionnaire was used to collect demographic and health data. Interviews were conducted in-person in the UK South West region, over Microsoft Teams and via telephone. All interviews were recorded, transcribed and analysed thematically for trends and meaning.

**Results:**

Five key themes were identified: AMR lack of familiarity, a low motivation to change long-term diet to reduce AMR, life enjoyment, prioritising general diet behaviours in lieu of AMR management, age-related barriers to behavioural change and healthcare and public-led diet-related AMR management. A low awareness of AMR generally appeared to solidify apathy towards the subject and a reluctance to consider any long-term dietary change for the purpose of AMR management. Comparatively, the cohort held strong opinions towards diet as a means of maintaining health and health decision-making. Independent diet management strategies were preferable over group sessions with the credibility of advice deemed crucial.

**Discussion:**

The older public’s unfamiliarity with AMR, antibiotics and the influence of diet demonstrates an urgent need for public awareness and education as a preliminary and essential action. Shifting the focus of AMR from an individualistic problem to a collective issue where we need to minimise the harm to others should be considered to inspire public motivation towards this important public health issue. The development of a dietary-related AMR management tool that offers tailored and strategic advice to a public population who already exhibit an existing interest in general dietary health is further recommended.

**Supplementary Information:**

The online version contains supplementary material available at 10.1186/s12889-025-25375-4.

## Introduction

### A global threat

Antimicrobial resistance (AMR), the process of microbes such as bacteria, fungi and viruses evolving to become resistant to the therapeutic effects of antimicrobials used to eradicate them, has been ranked by the World Health Organization (WHO) as one of the top ten threats to global public health [1]. Recent data collected as part of the Global Research on Antimicrobial Resistance (GRAM) project suggests that, in 2019, 4.95 million deaths were associated with AMR, of which 1.27 million were attributable to bacterial AMR [2]. The findings reinforce those of the O’Neill report, projecting 10 million AMR-related deaths every year by 2050, equivalent to one death every three seconds [3]. The global burden of AMR is considerable, it poses a significant threat to human life and requires a diverse and coordinated action plan [4–6]. There is a significant and urgent need to research and better understand the driving factors of human behaviour in AMR to provide the evidence necessary to prevent an avoidable and sizeable loss of human life.

AMR is a particular concern for vulnerable populations, such as children, older people and immunocompromised individuals [7]. Older people are more likely to experience poor health and have a weaker immune system and, consequently, the majority of antibiotic prescriptions in the UK are provided to those aged 65 years and over [8]. Likewise, the older population are associated with more frequent hospital admissions, exposing them to healthcare-acquired infections, and may receive devices in hospital, such as indwelling catheters, increasing the likelihood of developing an infection further [9]. Accordingly, older people pose an important target population for potential interventions. There is a strong rationale to explore AMR perceptions and health behaviours among the older population, who are more likely to contribute to the likelihood of resistant infections and be more severely affected by their outcomes.

### The role of diet in AMR

If microbes are exposed to antibiotics, then the eventual evolution of resistance seems to be mostly inevitable [10]. However, there are various factors that exacerbate the likelihood of resistant strains developing, from inopportune antimicrobial selection to antimicrobial misuse and poor sanitation [11]. As antibiotic resistance is encoded in the microbiome, diet may play a key role in our fight against AMR [12]. Interventions that seek to alter the taxonomic composition of the gut may allow a shift towards a microbiome containing fewer antibiotic resistant genes (ARGs) as evidence suggests that the long-term consumption of a diverse range of foods place selective pressure on AMR [12]. For example, the overuse of antibiotics as growth promotors, prophylactics, and therapeutic agents in aquaculture and food animal production has been shown to increase the likelihood of antimicrobial resistant bacterial transfer to humans [13], potentially increasing the reservoir of gut ARGs in animal-related diets. Raw meat, seafood and certain dairy products are associated with a medium to high potential for exposure to resistant bacteria [14], while a high pork and chicken intake is also thought to play a role in ciprofloxacin-resistant urinary tract infections [15]. Likewise, supermarket fruit and vegetables may also be sources of AMR if not properly washed with water before consumption [16], and ready-to-eat salad has been associated with ARGs, particularly proteobacteria, bacteroidetes, actinobacteria, and firmicutes [17].

The resultant ARG reservoir may contribute to AMR morbidity and mortality as resistant bacteria can translocate through the gut, spreading resistant infections throughout the body [18]. Likewise, resistant bacteria in the gut may transfer to healthcare staff via contact with faeces, causing healthcare-acquired infections among patients and inducing outbreaks of new resistant strains [18]. With diet an influential factor in AMR, it follows that changing health behaviours in diet may be a potential means of reducing the associated health burden. For dietary changes to impact AMR on a wide scale, an equally widespread commitment to dietary change would be required, potentially from the food industry, government and public themselves. However, research studies to date have ignored the human psychosocial and motivation elements associated with diet-related AMR management. Individual behaviours are necessary to drive change in a systemic approach.

Previous qualitative studies in this field have focused on meat producers’ and animal health practitioner perceptions of antimicrobial use and resistance [19–21]. The reported severity of AMR as a health issue was mixed among participants working in the food industry, yet studies consistently raise that additional awareness of the contributing factors of AMR, education and community engagement are essential [19–21]. Alternative diet-focused research that aimed to understand how consumers make sense of information about agricultural antibiotic use and AMR emphasise that concern for AMR is high but knowledge surrounding the link between diet and AMR is poor [22]. A similar focus group study suggests that members of the adolescent public lacked knowledge on the consequences of diet on the microbiome and intention to use antibiotics differed by level of scientific study [23].

This study sought to build on this evidence by aiming to better understand the older public’s attitudes towards shifting diet for AMR purposes. No research study to date has explored older people’s perceptions of diet-related AMR and their openness towards associated strategies. Resistant genes in the gut are more frequent in obese individuals [24]; being overweight or obese is more common in all age groups above 45 years, and people aged 65–74 years are most likely to be obese [25]. Thus, the older population are more likely to be exposed to the relationship between obesity and AMR risk, posing a potentially important target audience for interventions in this field – therefore, we were particularly interested in understanding perspectives from individuals who may be overweight or obese.

### Aim and objectives

The aim of this study was to explore older people’s perceptions of diet and health factors surrounding AMR. The following objectives were set:

- To examine barriers and facilitators towards people managing their individual AMR risk.
- To understand how people would perceive a diet-related AMR management tool.

Rather than gauge perceptions towards specific dietary changes, this study sought to explore perspectives towards *any* potential dietary change to better appraise the considerations taken to general diet decisions in AMR, everyday priorities, and individual considerations towards food groups. Consequently, this study has added to the field’s understanding of dietary behaviours in infection prevention; the broader approach taken should inform the direction of specific dietary interventions in reflection to participants’ preferences and described behaviours. Likewise, as an understanding and pre-existing commitment to AMR is thought to be key in driving positive AMR-related behaviours [26], this study has attempted to appraise participants’ openness to engaging in diet-related AMR management. This is important to future work in public-focused AMR management strategies and developing approaches digestible to their target audience.

## Method

### Study design

A qualitative study design was used. This research method was selected and justified as the study sought to collect rich, individual data about participants’ experiences and attitudes towards AMR and diet; this would not have been as effectively gleaned through quantitative or alternative qualitative methods [27].

### Study setting/context

This study was conducted in the Southwest region of the United Kingdom with all media recruitment focused on Bath, Bristol and the surrounding areas. This was practically necessary as face-to-face interviews were offered in all cases. 

### Study population

This study targeted older people who were more likely to both be obese and be prescribed antibiotics [8], and, therefore, would theoretically provide richer interview data surrounding antibiotics whilst also being at higher risk of AMR consequences. Participants were eligible to take part if they were aged 65 years or over, identified as a member of the public (healthcare workers, past or present, were excluded), were UK-based and fluent in English. A purposive sampling strategy was applied to source a diverse participant pool that was likely to be representative of the target population in relation to age, gender and ethnicity.

### Participant recruitment

Social and community groups relevant to the target population were contacted through an existing list of ‘gatekeepers’ associated with the university of Bath and online searches. Recruitment continued to take place until at least 15 participants had been sourced and interviewed. The target sample size for this project was estimated at 15 participants. This number of participants was guided and justified as suitable to gain ‘information power’ [28], and is similar to the sample sizes used in previous akin research studies [26, 29, 30]. Likewise, participants were recruited and interviews continued until data saturation was reached. Forty-three groups were contacted using the University of Bath’s Healthy Later Living network gatekeeper list. An additional 122 groups or establishments were identified by the researcher and contacted before the target of 15 participants was reached. All interested individuals (who responded to the recruitment material/email or poster) were initially contacted by the researcher (LF) via email or phone. They were then emailed or posted a participant information sheet and demographic questionnaire. All participants who met the inclusion criteria were invited to take part. Participants were asked to follow a link to an online questionnaire (or this was posted to them alongside a printed participant information sheet if they preferred), which had been created using the Qualtrics XM platform.

Following a higher response rate of White participants with BMIs that fall within the typical range from high socioeconomic backgrounds, recruitment was targeted to source a stratified sample following an initial eight interviews. For example, more deprived areas of Bristol were identified using Bristol City Council data [31], postcodes for these areas were cross-referenced with Slimming World sites in those areas and those sites were contacted with recruitment material.

### Data collection

Data was collected between July and November 2022. Initial demographic data was collected using self-administered questionnaires followed by either an in person or virtual interview (over the telephone or using Microsoft Teams).

### Questionnaires

Six of the questionnaires were completed on paper, either before the interview or sent in advance via post; the remaining nine questionnaires were completed on the online Qualtrics system. The demographic questionnaire collected informed written consent, basic demographic data including gender, age and postcode, highest level of education completed, smoking status and frequency of regular exercise. Additionally, height and weight data, existing health conditions and regular medications were recorded. The participants were also asked to select time slots that are usually convenient for their schedule so that an interview time may be arranged. Interview slots were offered seven days a week, between 8am and 8pm, to avoid selection bias associated with only allowing those with certain shift or daily activity patterns to take part [32].

### Interviews

Both in-person (at the University of Bath or a local coffee shop) and virtual interviews (over the telephone or Microsoft Teams) were conducted with all study participants, depending on what suited the participant best. The interview discussion focused on the participants’ self-perceived understanding of AMR, optimal antibiotic use, infection control behaviours, optimal diets, dietary interventions and attitudes towards managing diet for AMR priorities. A full topic guide can be found in Additional File 1 and all participants were debriefed at the end of each interview. Once a convenient time had been arranged, all interviews were conducted on a one-to-one basis. A mixture of interview environments were used to reduce bias associated with older people who were more or less technologically able [33], or limiting public contact due to infectious disease risk, increasing study uptake. All participating individuals received a £10 ‘Love to Shop’ gift card in compensation for their time.

Five of the interviews were completed face-to-face, either at the university or at a local coffee shop, seven were conducted by telephone and five were completed over Microsoft Teams. Of the five meetings that were conducted over Microsoft Teams, one participant chose to turn on their camera. The mean length of interviews was 46 min (with a range of 30 to 78 min).

### Data analysis

Interviews were recorded using Microsoft Teams and its automatic transcription software; all automated transcripts were checked for accuracy, errors corrected and anonymised prior to analysis.

Reflexive thematic analysis of the transcribed data was conducted based on Braun and Clarke’s practical approach [34]. The flexible application of reflexive thematic analysis allowed the researcher to develop themes pertaining to the factors that influence, underpin or contextualise older people’s behavioural motivations in diet and antimicrobial use. The process involved familiarisation with the data, by relistening to the recorded interviews and re-reading the transcripts until the researcher was familiar with them in their entirety [27]. Using NVivo software, codes were developed that captured specific and particular meanings within the dataset [34]. A hierarchy of codes was built within NVivo that reflected the different domains and sub-themes within the data. The dataset was reviewed thoroughly twice and the code labels were refined, finalised and checked for consistency [34]. Similarities in meaning between the codes were considered, clustered and connected in the development of themes [34]. Thematic maps were developed in NVivo to consider how provisional themes related to each other and the overall research question before themes were refined, defined and conclusively named.

Reflexive Thematic Analysis Reporting Guidelines (RTARG; see Supplementary Materials) were followed to encourage a values-based approach that aligned methodological coherence with high quality reporting of the thematic analysis [35]. The RTARG consists of advice for reporting reflexive thematic analysis, guiding notes and common errors to be avoided. For example, the guidelines advise researchers to ‘Describe selection of participants’ and ‘Discuss how the researcher(s) engaged with the analytic process’ [35]. Each relevant guideline was considered and actioned during the reporting process.

### Ethical considerations

The study received favourable opinion from the Psychology Research Ethics Committee (PREC) at the University of Bath (reference number 22 101). Data was collected in accordance with the Declaration of Helsinki [36]. All participants provided written and verbal consent prior to taking part in this study. For ethical purposes, participants had at least 24 hours between completing the informed consent form and questionnaire and their interview slot. Written consent was gained through a PREC approved consent form, which was provided to participants at the start of the online questionnaire. All participants were encouraged to raise any queries at the point of email recruitment and verbally before and after the interview. A follow-up debrief was additionally offered to all participants.

## Results

### Participant summary

In total, 17 older members of the public were interviewed as part of this qualitative investigation. The participant pool had a fairly even gender split (eight males and nine females) with the majority identifying as White (*n* = 13) and completing post-secondary school vocational or university education (*n* = 14). Likewise, there was a diverse breadth of ages seen, with the 65–70 category the most common with eight participants. A full breakdown of participant demographic and health data is shown in Table 1. Additionally, information relating to any diagnosed health conditions and relevant medications was also gathered. BMI data was collected; however, reports suggest that BMI measures show a minimal relationship to mortality risk and poorly identifies obesity in older people [37]. Additionally, the participants’ postcodes were used to calculate deprivation using the English indices of deprivation tool [38].

**Table 1:** Participants’ demographic characteristics and health measures (exercise regularity, smoking status, recent infection history and BMI category). n = number of participants

	*n*
**Gender**	
Male	8
Female	9
Non-binary/third gender	0
**Prefer not to say**	0
**Ethnicity**	
White	13
Black, African, Caribbean or Black British	3
Asian/Asian British	0
Mixed/Multiple ethnic groups	0
Other	1
**Age Group**	
65–70	8
71–75	4
76–80	1
81–85	2
86–90	1
91+	1
**Highest Level of Education**	
No schooling completed	0
Secondary school graduate	3
Professional/Vocational/Technical Training	6
Bachelor’s degree	4
Master’s degree	3
Doctorate degree	1
**Deprivation**	
Multiple Deprivation Decile range: 3–10 (median 7)	-
Income Deprivation Affecting Older People Index range: 1–10 (median 8)	-
**Frequency of engaging in exercise lasting longer than 30 min**	
Less than 1x per month	3
Once a month	1
Once a week	3
Two/three times a week	3
Four/five times a week	3
More than five times a week	4
**Smoking status**	
Smoker	2
Non-smoker	15
**Bacterial or viral infection during the two weeks prior to questionnaire completion**	
Yes	3
No	14
**BMI Category**	
Underweight (< 18.5)	1
Healthy Weight (18.5–25)	10
Overweight (25–30)	2
Obese (> 30)	4
**Self-reported duration since last taking antibiotics**	
Can’t remember	4
Last 12 months	4
Last 1–5 years	5
Last 5–10 years	1
Over 10 years	3

Following thematic analysis, five core themes were developed. These were AMR lack of familiarity, low motivation to change long-term diet to reduce AMR, life enjoyment, prioritising general diet behaviours in lieu of AMR management and healthcare and public led diet-related AMR management. For a full list of themes and sub-themes, see Table 2. Additional observed barriers to general health behaviour change in diet across the cohort included food costs, what was easy to prepare, inclination, living alone, taste and time.

**Table 2:** Themes and sub-themes developed through the thematic analysis

Theme	Sub-theme
AMR lack of familiarity	-
Low motivation to change long-term diet to reduce AMR	Emotional Indifference Towards AMR
	Reluctance to prioritise dietary change
	Reluctance to engage long-term
Life Enjoyment	Life is short: A reluctance to compromise
	Optimism bias
Prioritising General Diet Behaviours in Lieu of AMR Prevention	-
Intrinsic and Extrinsic Change Motivation	Intrinsic motivation
	Doctor-led motivation

### AMR lack of familiarity

Participants described varying levels of AMR understanding and awareness with most participants not aware of the term ‘antimicrobial resistance’ and some participants unsure of what antibiotics were. This is relevant as an awareness of AMR is pinnacle to an understanding of diet-related AMR management.

*Yeah*,* it [AMR] is because it’s something I don’t really know anything about…. Particularly unlike things like climate change for they’re in your face all the time (Participant 12)*.

In all cases, participants described not having an awareness of a link between diet and AMR, instead often attributing the cause of AMR to perceptions of practitioner overprescribing and patient overconsumption of antibiotics. Likewise, most of the participants described the type or abundance of antibiotics having changed across their lifetime, and suggested that AMR did not exist when they were younger. Further common assumptions were that resistance was associated with the functioning of the body rather than the microbe, that ‘*when you are getting to different stages of life*,* you need different types of antibiotics’* (Participant 14) and an overarching belief that AMR did and would not affect them as they individually did not take a lot of antibiotics. Comparatively, in a few cases, concern was present among the higher educated individuals and, understandably, those from science backgrounds, with Participant 1, a PhD educated individual, describing the situation as ‘*quite scary’*.

### Low motivation to change diet to reduce AMR

Across the cohort, participants described a low motivation to change their diet to reduce AMR. The individual reasons for this varied but centred around an emotional indifference towards the issue, a reluctance to prioritise dietary change in a perceived disruption of a ‘status quo’ and a low motivation to invest long-term.

#### Emotional indifference towards AMR

Overall, across the group, the emotional concern for and perceived risk of AMR was absent and this was largely connected with poor knowledge surrounding AMR and diet-related AMR management. In all transcripts, this emotional indifference led to reduced motivation to act.

*It’s not something I give that much thought to. Uh*,* I know that doctors have been accused of prescribing too many. That’s about the only thing I know about antibiotics… It hasn’t had any effect on my life or my behaviour or my anxieties or anything.(Participant 8)*

Rather than a subject that participants had been exposed to, considered and consciously decided to approach with low concern, AMR attitudes were often associated with a longstanding emotional indifference.

*It’s not something I’m worried about. You know*,* it’s sort of not at the forefront of my worries. (Participant 12)*

Participants would report an emotional indifference towards the subject in tandem with self-perceived beliefs that AMR was something that ‘*doesn’t really affect me’* (Participant 3). As pinpointed, a personal lack of concern for AMR was described with the erroneous belief that drug resistance was to do with the person themselves being resistant and not the microbe. This was posed as a concept that, as a self-perceived healthy individual, they need not be concerned about, resulting in a distancing from AMR as something that is not worth emotional or behavioural investment.

#### Reluctance to prioritise dietary change

When participants were probed for attitudes towards hypothetically changing diet to support AMR efforts, a predominant barrier to dietary behavioural change was a distinct reluctance to do so, steeped in scepticism towards AMR-related dietary advice. Only four participants replied that they would be open to changing their diet for this purpose.

*I’d take the advice. I’d try it. I’d give it a go. If I really*,* really had to*,* for my health*,* to keep going. (Participant 11)*

Thus, even in the case of Participant 11, who was open to changing their diet, their decision was shrouded in reluctance and as a means of strictly necessary health management. Comparatively, the group reported that their general motivations for changing diet stemmed around weight-loss, disease-management and not wanting ‘*to end up in hospital or end up having more tablets’* (Participant 13).

*And so I don’t want. I don’t want to be worrying anymore about it [Diet] more than I have to. (Participant 8)*

#### Reluctance to engage long-term

Participants did report being open to changing diet in the short-term (for the duration of an antibiotics course) if they felt it would improve the efficacy of the medication, with abstaining from alcohol while taking antibiotics a common example. This behaviour stemmed from a reluctance to unnecessarily ‘*suffer’* (Participant 9) and being unsure why they would need to change their diet if they were not ill with an infection.

*Well*,* if it [changing diet] would have effect on the the efficiency*,* if you like*,* if I had to take an antibiotic. Then of course I would do it*,* but only for the duration of the course. If you see what I mean… The antibiotics cure the infection*,* once the infections cured*,* what’s the point in carrying on treating it? (Participant 10)*

Participants were willing to change their diet for the duration of an antimicrobial drug course, but not in a long-term preventative approach to AMR. Equally, the perceived scale of the dietary sacrifice was commonly raised as a consideration.

*You know*,* if they tell me to cut down on one particular food*,* then that’s easy. If you tell me to cut out meat*,* that would be impossible. (Participant 15)*

Thus, the change needs to be perceived as achievable, acceptable and realistic if the population are to attempt behavioural change in AMR.

### Life enjoyment

Life enjoyment and a perception that changing diet for AMR purposes meant sacrificing a daily joy fed a distinct reluctance to be open to diet management strategies. This reluctance stemmed from a perception that life is finite and should therefore be enjoyed. Such views appeared alongside perceptions that the sacrifice to life enjoyment does not seem justified for a seemingly small risk given they have perceived themselves to be healthy so far.

#### Life is short: a reluctance to compromise

Participants portrayed changing diet as a highly tangible sacrifice that was not easily justified against a seemingly far-away and uncertain possibility of antimicrobials ceasing to be effective. This demonstrates how health decision-making and a reluctance to change is informed by weighing the health risk, investment and benefit. This in part arose from a perception that life is finite and therefore should be enjoyed.

*I think there’s too much noise on ‘eat this’ and ‘eat that’. Have what you like*,* life is too short. (Participant 11)*

*I mean I could [change diet to reduce AMR]. You know*,* I don’t deny that I probably drink more than I should*,* but I’ve got a life to live. (Participant 10)*

This was further reinforced with perceptions that, in a seemingly finite lifespan, the sacrifices required to make a difference in public issues were too great.

*I know for the sake of the climate*,* we should be eating less meat*,* but that doesn’t stop me eating meat. (Participant 8)*

In this study, an older population was used and this status of old age appeared to be linked with a heightened focus on health and morbidity generally, while age-related diagnoses were reasons not to change diet for unrelated AMR purposes. All participants perceived and described themselves as, overall, healthy people with good diets and a limited need for antibiotics. Yet, in many cases, medical conditions such as arthritis, hypertension, menopause-related food intolerances, reduced mobility and not being ‘*as fit and able as I was’* (Participant 11) were described as perceived barriers to engaging in diet-related AMR behavioural change. Equally, behavioural change, particularly when perceived as unnecessary, was described as a potential threat to a status quo of managed health conditions for limited future benefits.

*I would work on what tended to suit my own body. I’m not going to put my own body under real problems for the sake of some punitive benefit in the future. (Participant 2)*

A reluctance to be open to changes that may influence existing health management may be unique to the older population who are more likely to have co-morbidities and potentially demonstrates the limitations when approaching behavioural change in this population. Nevertheless, the concept of future benefits versus the individual behavioural cost appears to be important to decision-making in AMR.

#### Optimism bias

Participants’ responses demonstrated the power of personal gratification from food as a health behaviour motivator. This appeared to particularly be the case given the exposure of diet as an integral part of daily life; with this, dietary temptations appeared to bring regular fulfilment and happiness to the older population. Comparatively, exposure to antibiotics and AMR were significantly more limited with many participants reporting not having taken antibiotics for many years, rendering diet-related AMR approaches a less persuasive argument. This appeared to be reinforced by unrealistic optimism as a resistant infection hadn’t yet occurred in their longevity and was perceived to therefore be unlikely to happen in the future, with one participant reporting:

*Well*,* something must be working for [the participant’s spouse] and me. I mean*,* like I say*,* I’m 91. [The participant’s spouse] turned 80. So something’s working. I mean*,* I’m not saying we’re perfect*,* we’re not. But*,* whatever we’ve been doing so far*,* it’s working for us*,* isn’t it? (Participant 17)*

This demonstrates how Participant 17’s current dietary behaviours have positively reinforced protective beliefs over their current health behaviours and potentially a reluctance to change these in response to a threat that isn’t perceived to have affected them within their current health regime.

### Prioritising general diet behaviours in Lieu of AMR prevention

The concept of diet as a line of defence in disease prevention was a key motivator that drove the deprioritising of specific diet-related AMR strategies among the group. Comparatively, antibiotics were seen as required when a poor diet had contributed to disease.

*I put healthy diet first because*,* if your body is happy*,* if it is quite happy with what is going into it*,* on the whole*,* it shouldn’t need anything outside*,* so the artificial to help it on its way. (Participant 4)*

Participants described the prioritisation of diet in health behaviours in a whole-system approach to boosting their immune system and reducing the likelihood of requiring antibiotics and, consequently, lessening their risk of contributing to antibiotic or treatment complications.

*Because if you go on a healthy diet. Then your body will take what it needs from the diet and from what you’re eating*,* and it should keep you going. And [receiving] an antibiotic is because something’s failing. (Participant 5)*

This potentially demonstrates how diet is seen as a preliminary, preventative step to health consequences such as those associated with AMR and antibiotics the ‘patch’ when the initial line of defence becomes ineffective. Rather than dietary change in AMR strategy, this emphasises the public’s existing ranking of diet in disease prevention, as summarised by Participant 7.

*Well*,* diet is a permanent thing and antibiotics would only be priority if I had an infection that wasn’t going away naturally. (Participant 7)*

### Intrinsic and extrinsic change motivation

Across the cohort, participants described external sources of information or guidance and how these would influence their decision-making in diet-related AMR management. Perceptions varied between those with a strong intrinsic motivation, who preferred to drive their own health behaviours, and others who felt they would be more strongly influenced by health professional, specifically doctor, advice.

#### Intrinsic Motivation

Participants commonly responded negatively to the idea of diet-related AMR management strategies that restricted or instructed the public on what to eat, preferring public advocacy and education approaches.

*I won’t be told to eat something*,* I choose my own. (Participant 11) *

*I wouldn’t like to think that we were becoming a nanny state. (Participant 12)*

Additionally, participants described how they would prefer to do their own research rather than trust medical or national campaign advice before engaging in diet-related AMR management. As shown below, this stemmed around distrust towards the sources of advice.

*If it’s sensible*,* you gotta weigh it up. You don’t just take their [advice]. And what they’re [medical professionals or government campaigns] trying to get you to do. You do a little read around. (Participant 10)*

Likewise, many of the older participants remembered a post-war era and this was associated with a consumption culture. These views appeared to be derived from both war-time related parental input and government relaxing rationing restrictions.

*I’m of the generation where it was the end of rationing from the Second World War. And my parents were of the belief that if a plate of food was put in front of you*,* you ate all of it. (Participant 3)*

When asked if they would rather take part in an intervention independently or as part of public group sessions, most of the participants preferred to take part alone, five preferred group sessions and one participant had no preference. The cost of sessions, fear of COVID-19 spread in group environments and determining which routes were quickest were considerations in the cohort. Group sessions were thought to be beneficial as ‘*two heads are better than one’* (Participant 13), whereas independent routes were deemed preferential as ‘*I would hope that the intervention would be specific for me*’ (Participant 15).

#### Doctor-led motivation

Most participants placed doctor’s advice in high esteem when directing their health behaviours and particularly when it came to medications. Despite many participants being reluctant to take medication, all participants responded that they would still take a prescribed antibiotic even if they didn’t know what the drug was treating or agree that they needed an antibiotic. This choice hinged on a doctor’s accrued medical knowledge and trust in the profession.

*I like to think an antibiotic is a last resort. If she [the GP] said go and stand over in the corner*,* naked*,* on your head*,* and it would get rid of it [toothache] then I probably would have because that’s all I wanted. The majority of people would do because it’s the trust we have in the NHS. It’s*,* I don’t know how to put it*,* but*,* cradle to the grave. You go to see the doctor and you put the trust in them. (Participant 5)*

This trust in the credibility of health advice spanned outside of just antibiotic taking, with doctor advice important to general health behaviours, suggesting an important source of public direction in diet-related AMR interventions.

*“I think when it comes down to me*,* I would always trust them. Because A) I want to get better and B) they know more than I do”. (Participant 15)*

Additionally, Participant 5 compared the dietary advice received by doctors to the trust they had placed in surgeons when signing consent to medical procedures with unlikely but severe risks. However, in one case, this trust was matched with feelings of a reluctant loss of control.

*If they said I needed them them*,* you know*,* I*,* I*,* I’d take their word for it. They’re the professional… I don’t think you’ve got much choice have you? (Participant 10)*

This was tinged with Participant 10 further questioning the motives of the health professional, as shown below, demonstrating the importance of gaining public buy-in to any proposed changes.

*So when my doctor says ‘how many do you smoke and how much do you drink?’*,* I don’t tell him. It’s none of his business. They’re just doing a survey. (Participant 10)*

## Discussion

### Summary of findings

Findings from this study indicate that older persons’ knowledge, awareness and emotional concern for AMR was limited. Conversely, participants had a good understanding of what constitutes a healthy diet and considered diet important in health behaviour decision-making, although they lacked awareness of diet’s role in AMR. Motivating people to adopt diet-related AMR strategies appeared challenging due to scepticism and low motivation, particularly among less educated and lower socioeconomic groups.

Participants viewed diet as crucial for overall health but were reluctant to make long-term dietary changes to reduce AMR risks, placing more emphasis on short-term adjustments during antibiotic courses. This reflected a ‘life is short’ mentality, a view that they personally had avoided AMR so far. Finally, health behaviours were motivated both intrinsically and extrinsically; preference appeared to vary on an individual and subjective basis. Below we present a discussion and interpretation of the findings from this study in relation to existing literature.

### Knowledge and awareness as a preliminary step to AMR dietary behavioural change

Outside of a couple of educated exceptions, knowledge of AMR and antibiotics across the group was generally low. Views of AMR were often attributed to overprescribing and overconsumption of antibiotics. This is to be expected given the focus of national campaigns such as ‘Keep antibiotics working’ and the ‘Antibiotic Guardian Campaign’ on only seeking antibiotics when necessary [39, 40]. Comparatively, all participants held strong, informed and similar views surrounding what constituted a healthy diet. This finding reflects those of previous qualitative research, which suggest that public awareness and knowledge surrounding the role of diet and food in AMR is lacking [22, 23]. Equally, the concept of diet-related AMR management strategies was met with low motivation, scepticism and a desire to conduct personal research, demonstrating the importance of existing AMR awareness and education as an essential component for behaviour change [41, 42]. This study highlights that what is first required is engagement with the public to increase their awareness of AMR generally and diet-related AMR factors. This needs to be completed as a preliminary step or in conjunction with strategies seeking public-driven changes to diet for AMR purposes.

As this research found that the highest educated participants indicated a greater awareness of AMR, there is potentially a need to promote awareness among less educated populations and lower socioeconomic groups. Similar trends of misinformation are seen in less educated populations internationally [43], while lower socio-economic groups also pose one of the hardest populations for health campaigns to access [44]. Participants did not see themselves to be at risk of the consequences of AMR, potentially as a result of limited understanding of threat information, perceived argument quality or perceived source credibility. This supports previously reported recommendations, which endorse effective risk communication through targeted message framing, proactive, cross-sector One Health Awareness campaigns and an antibiotic food labelling system [22]. Media saturation using posters, GP surgery leaflets and television adverts that direct the public towards a dietary AMR assessment and advice resource could be beneficial. There needs to be a shift from AMR being presented as an individualistic problem of optimism bias to framing the issue as one that expands the sphere of responsibility [45].

### Motivation to invest in diet-related AMR management

#### Existing investment in diet

The results demonstrated that participants had a clear view of ‘healthy foods’, considering diet to be an important component to a healthy life, as previously identified in older person research [46], and a ‘first line of defence’ in infectious disease. There is merit to this belief, with diet associated with antioxidative status and functioning of the immune system [47, 48] and infection prevention is key among existing AMR initiatives [49]. However, these beliefs were very generalised towards infectious disease and not resistant infections further demonstrating the challenges of changing health behaviours without first raising awareness.

This finding can in part be explained due to a lack of self-perceived diet-related AMR management knowledge among this sample of the general public. The population’s existing interest in diet could be harnessed if current awareness were to expand into a greater appreciation of AMR and diet-related AMR management. This raises the rationale for greater awareness and knowledge campaigns that promote discussion and engagement within the public realm.

#### Short-term sacrifice for short-term benefits

Despite the perceived link between diet and health being clear across the group, with variable specifics, participants were reluctant to theoretically change their long-term diet to reduce the risk of AMR. Individuals predominately only showed a willingness to alter diet for the duration of an antibiotics course, a short-term sacrifice for a short-term incentive. This hints at a lack of understanding of how dietary factors may influence the risk of AMR. Likewise, this demonstrates the influence that health incentives and the perceived risk of disease play in diet-related decision-making. As gut-related AMR infections are largely a *result* of close contact with associated livestock or consumption of a contaminated food source [18], dietary change at the point of infection and antibiotics is largely unrelated to the risk of resistance.

The finding demonstrates that in order for the public to change their diet, the perceived health benefit must outweigh the perceived sacrifice of the lifestyle change. This finding is reflected in the Health Belief Model [50], with the framework considering perceived susceptibility. A dietary change cannot be seen to significantly reduce life enjoyment and ideally could be framed as part of a life enjoyment approach to be effective, or if it is perceived to do so then the risk of resistant disease and benefits of avoiding such outcomes must outweigh this.

Low motivation to invest in diet-related AMR management appeared to particularly be the case given the regular exposure of diet as an integral part of daily life compared with a faraway and less tangible concept of untreatable infections. The qualitative work supports the low perceived behavioural control over AMR and dispositional pessimism as influential roots of susceptibility beliefs [51]. Increasing the tangibility of dietary health risks in AMR could serve to shift the public’s outcome expectancies, as described in social cognition theory [52]. This could be achieved through relatability and personalised messaging, a strategy previously used in the Department of Health and Social Care’s sharing of ‘COVID’ stories [53].

### Tailored approaches to diet-driven AMR management

Participants preferred two sub-themes of somewhat contradictory approaches to diet-related AMR management. On the one hand, participants felt motivated to seek out their own information on diet and health, rejecting the idea of health instruction. Comparatively, a cluster of NHS support and unwavering trust in health professional’s advice was observed. These sub-themes may seem somewhat contradictory, a characteristic of thematic analysis, which is not necessarily to tell a uniform, singular narrative but convey the evidently patterned meaning [34]. The spectrum of preferred approaches could be due to individual personality, with those favouring independent strategies harbouring a high degree of perceived behavioural control in diet and AMR [50]. Comparatively, it is thought that individual circumstance is influential, with older men in particular at risk of poor dietary management in health and more likely to seek help from external sources than manage independently [54]. Likewise, the trust placed in doctors’ advice has been well documented [55] and solidifies healthcare providers as a credible source in this field.

Healthcare-led and community approaches alike should be used to build a subjective norm of prosocial behaviours in diet-related AMR management, where the positive intent to modify diet for AMR purposes is underpinned by social compliance. Participants’ scepticism and preference to change their diet for weight loss, disease management or antimicrobial efficacy purposes creates a complex parallel in AMR, an issue requiring collective action, when comparing behavioural intention for personal gain versus altruistic intentions. Drawing on lessons from climate change, a similar issue requiring widespread individual action for a collective cause, a high level of awareness and acceptance is needed to instigate social change [56]. Participants’ self-driven motivation highlights the importance of cooperation and conformity as social drivers; the public would be more likely to do their part if they know others are doing theirs [56, 57].

This study found that, in the older population, some opinions of diet stemmed from post-wartime experiences, where a similar wartime spirit and fight against bacterial resistance could be communicated in diet-related AMR management strategies. It is theorised that an association may exist between national food restrictions and a time of hardship that individuals would be wary to return to, potentially curbing investment in similar widescale dietary approaches in AMR; however, this is hypothesis only and further research would be required to explore this. In a similar way that campaigns have centred on widespread vaccination to protect the ‘vulnerable’ and banned smoking from public areas to protect others from passive smoking [58, 59], diet-related AMR management strategies need to reframe the problem and nurture the public’s appeal not to harm others. In light of fears of a ‘*nanny state’* (Participant 12), appealing to the collective protection of individual rights to access resistant infection treatments may be enough to overcome protests of paternalism in diet-related AMR management.

### Study strengths and limitations

This study has provided insight into the real-world perceptions of the risk of AMR among older members of the UK public. It has also demonstrated how they perceive personal acceptability of potential dietary interventions. Both outlooks have not been adequately captured in previously published research. Data saturation was reached, allowing for a comprehensive analysis of perceptions. Likewise, it is thought that the integrity and reliability of this study’s findings were fostered through a congruent approach to participant communications, interviews, and analysis.

As has been previously reported [60], recruitment of an older population appeared challenging. The recruitment strategy used may have been prone to volunteer bias, a particular issue in dietary research where those who already held a strong interest and positive behaviours in diet may have been more likely to volunteer for associated research [61]. Likewise, demographic factors such as BMI and the duration since last receiving antibiotics were self-reported, thus reporting may have been affected by recall bias [62]. By extension, social desirability and recall bias are potential limitations, as participants may have been more likely to remember instances of positive diet and antibiotic use [63]. A greater proportion of 60–70 year olds were recruited and this may have influenced findings. Nevertheless, this age group finding is to be expected given life expectancy reducing available participants in older categories and given that younger elderly people are more likely to visit and engage with the community groups that were largely contacted during the recruitment of this study [64].

A variety of interview options (face-to-face, telephone or Microsoft Team environments) were offered to participants to increase the ease of taking part and avoid biases associated with mobility and technology-literacy. However, this may have caused inhomogeneity in the participant pool as social cues can vary between online and face-to-face contexts [65]. The validity of the data collected could have been increased with follow-up ‘validation interviews’ to solidify what is known about the participants’ core beliefs [66]. Finally, as only one researcher conducted the interviews and thematic analysis, there is a risk of researcher bias if the verbatim data was analysed subjectively [67].

## Conclusion

Older people’s perceptions of the role of diet in AMR management are complex and multi-faceted. Participants were very informed about the role of diet in health, but largely unaware of the role of diet in AMR. This was commonly associated with apathy towards the subject and dietary change presumed to be too large a sacrifice to daily life for seemingly punitive benefits to infection management. In all cases, the credibility of advice was understandably vital to individuals’ openness to dietary change, but this was hindered by diet being seen just as an action for personal gain. Resultantly, public awareness of AMR is an essential and preliminary step to any further behavioural change. Policy-makers and campaign leaders need to focus on reframing the issue of AMR from an individualistic to a collective problem which requires widespread individual action. In particular, communicating diet as a preventative rather than reactive action that protects the wider public outside of one’s self will be vital to acknowledging the relationship between AMR, social change, public awareness and human health.

## Supplementary Information

Supplementary Material 1.

Supplementary Material 2.

## Data Availability

The datasets used and/or analysed during the current study are available from the corresponding author on reasonable request.
