# Supplementary material for: A qualitative evaluation of older people’s perceptions towards optimal diet management in the context of antimicrobial resistance
Source: BMC Public Health. 2025 Nov 19;25:4060. doi: 10.1186/s12889-025-25375-4 (PMC12628876; doi:10.1186/s12889-025-25375-4)
Supplement: Supplementary file 1 — Supplementary Material 1. [file 12889_2025_25375_MOESM1_ESM.docx]

## Topic Guide

| **Topic** | **Aim** | **Example Questions** |
| --- | --- | --- |
| Antibiotic history | To determine antibiotic history | - When was the last time you took antibiotics? - How many times have you taken antibiotics in the last year? - [If listed supplements in preliminary questionnaire] Talk me through why you decided to start taking supplements. |
| Participant understanding of AMR | To examine participants’ knowledge of AMR | - Overall, what is your knowledge of antimicrobial resistance or antibiotic resistance? - [If an understanding of AMR] Where has your knowledge of AMR come from? [Formal education/GP advice/ Self-motivated learning] - What are the risks of taking antibiotics/antimicrobials incorrectly? |
| Infection control behaviours | To explore behaviours relating to infection control and prevention | - Talk me through at what point you would go and see a doctor or other health professional with an infection, such as a chest infection or stomach bug? - Which type of health professional would you choose to see in the first instance? [Non-prescribing Pharmacist vs. doctor etc.] - Has your need for antibiotics or your reliance on them changed as you have gotten older? - Do you feel that younger members of your family or friends reflect your same attitudes towards antibiotics? - Have your opinions towards medicines used to treat infection changed as a result of the COVID-19 pandemic? |
| Diet perceptions | To explore perceptions of a healthy diet | - Tell me what a healthy diet means to you? - What (if anything) makes it difficult for you to maintain a healthy diet? - Do you feel the need to maintain a healthy lifestyle has changed as you have gotten older? |
| Dietary change behaviours/ motivations | To explore attitudes towards dietary intervention | - Have you tried to alter you diet for health reasons over the last year? - Have you been made aware of any dietary interventions, such as reducing the volume of food you eat or the keto diet? - If yes, who told you about this intervention? [Friend/GP/Media] - What barriers or challenges did you encounter? / What barriers or challenges would you anticipate encountering? - What could motivate you to follow a healthier lifestyle? [Poor health risk vs. health benefits] - Would you prefer to engage in a dietary intervention scheme alone or in a group? - Describe your approach to exercise. |
|  | To appraise older peoples’ perceptions when balancing lifestyle and AMR priorities | - How important do you see antibiotics and other infection medications to your health compared to maintaining a good diet? - How do you feel about the idea of following a dietary intervention to reduce the risk of antimicrobial resistance? - Would you change your diet to reduce AMR, even if you had a healthy weight? - Would you be more likely to follow a dietary intervention if it was likely to keep antibiotics effective for when you specifically need them or if it maintained their effectiveness on a wider public scale? |
