# Supplementary material for: A qualitative evaluation of older people’s perceptions towards optimal diet management in the context of antimicrobial resistance
Source: BMC Public Health. 2025 Nov 19;25:4060. doi: 10.1186/s12889-025-25375-4 (PMC12628876; doi:10.1186/s12889-025-25375-4)
Supplement: Supplementary file 2 — Supplementary Material 2. [file 12889_2025_25375_MOESM2_ESM.docx]

# Appendix 13 - Reflexive Thematic Analysis Reporting Guidelines (RTARG)

*Based on the work of Virginia Braun & Victoria Clarke (2024) (columns 1 - 3) with reflective comments relating to this study in column 4.*

| **Advice for aspects of the research report/approach to reporting** | **Guiding notes and further explanation** | | **Practices, concepts and terminology to avoid** | **Author comments** |
| --- | --- | --- | --- | --- |
| *Background and rationale* | | | |  |
| Provide a robust context and rationale for the proposed research in the Introduction. | Can discuss existing research, theory, and the wider context; the researcher is understood as entering a conversation with existing scholarship. | | Critiquing the methodological limitations of existing research from a (post)positivist/ quantitative standpoint; orienting a literature review to finding a “gap” that the  research fills. | The Introduction section provides a background to the relevant contributing factors in AMR and the relevance of the target population, providing context for the study’s rationale. The existing body of research examining the influence of diet in AMR is scant with qualitative data exploring public perceptions towards AMR dominated by antibiotic-taking behaviour research. While the value of previous approaches is acknowledged, this paper was necessary to better understand the older public’s attitudes towards shifting diet for AMR purposes. This study does not seek to find a gap to fill but rather focuses on how it is related to existing scholarship in this area. |
| Clearly articulate a research question – one that is methodologically coherent. | Can discuss refining an initially broader research question to a more specific one for the  paper. | | Formulating research questions as hypotheses or expectations about what might be “found”. | The research aim and objectives are explained at the end of the Introduction, with a designated section. The reporting of results has been structured in a way that links and refers back to the aim and objectives. The objectives do not speculate what may be found but are framed around potential inputs and manifestations of participant behaviour. |
| ***“Owning your perspectives”*** | | | |  |
| Include information on guiding theoretical assumptions and  other (e.g., explanatory) theory informing the use of  TA. | Guiding (e.g., paradigmatic, ontological and  epistemological) and other theory should be coherent  with RTA. | | (Post)positivism and (simple) realism. | The Method section highlights the philosophical perspective of the study, which is then reflected throughout the discussion of the analysis and findings. The thematic analysis used is based on the reflexive approach used by Braun and Clarke. It was informed by an essentialist/realist approach, which was selected to theorise motivations, experience and meaning as it related to language during the analysis. |
| Report in a way that is consistent with stated theoretical assumptions throughout. | Theoretical coherence is evidenced through the use of language and concepts (e.g., around theme development, research subjectivity, data interpretation), the treatment of data, and use of quality  practices consistent with RTA. | | Inadvertently “mashing-up” of RTA and (post)positivism/ realism (e.g., assuming data interpretation can be accurate and reliable) – without a clear rationale. | The reflexive thematic analysis approach has been employed throughout the study and care taken to report accordingly. Consideration was taken when determining and reporting the methods used to distance from positivist terms such as reliability and objectivity. |
| Evidence methodological coherence/integrity in both the research and the report. | Theoretical assumptions, research questions, methods/practices of data generation, RTA, and specific orientation to RTA, purpose of research etc. all “fit together”,  conceptually. | | Ontological and epistemological confusion (e.g., claiming constructionism but focusing on lived experience and treating language as a transparent window onto this). | The method section discusses the reflective thematic analysis approach taken, its appropriateness in this study and how the analysis was underpinned by an essentialist approach. The specific research question, analysis and code generation were considered as a whole concept and analysed seamlessly with derived meaning. |
| Show evidence of reflexive practice. | Can discuss researcher professional or personal positioning and experience in relation to the topic, and/or participant group, and/or their role in shaping the research;  use of reflexive journaling. | | Evoking researcher bias (positivist), or even researcher influence, in a way that evokes it as possible rather than inevitable. | First-person accounts were not included as they did not reflect the third-person style of journal writing. |
| Write in a methodologically coherent style. | A first-person writing style suits RTA, as it “writes in” the researcher and contributes to situated and reflexive  reporting. | | A third person writing style – writing the researcher out of the research. | A third-person writing style was followed as per journal writing conventions. However, the role of the researcher has been highlighted throughout the Method section, from the selection of participants to participant communications, the determination of thematic analysis codes and limitations of one researcher completing the full analysis. |
|  | |  |  |  |
|  | |  |  |  |
| Describe selection of participants/data items. | Should include criteria for selection and/or recruitment strategies and settings. | | Terms “sample/sampling”, which connote “sampling” from a population (for the purpose  of statistical generalisation). | Participant selection is discussed in detail in the Method section, which outlines the selection criteria and community group recruitment strategy. Likewise, the study setting (UK South West) has been documented with justification. |
| Describe number of participants/data items; provide a rationale or explanation around dataset or participant group size/composition. | Non-positivist qualitative concepts, such as “information power” or sufficiency offer conceptually appropriate justifications for “dataset” or “participant group” size and composition. | | Justification based on saturation (simple realist), or statistical models (positivist); reporting rates of non- participation (an indicator of the representativeness of the “sample” in quantitative  research). | The number of participants and its adequacy are discussed in the ‘Participant Recruitment’ section. It is noted that the number of participants was guided and justified as suitable to gain ‘information power’, and is similar to the sample sizes used in previous akin research studies. Likewise, participants were recruited and interviews continued until data saturation was reached, as is consistent with the reflexive thematic analysis approach. |
| Discuss characteristics of participants/data items. | Balance the need to “situate the participant group” with participant anonymity (e.g., aggregate or report minimal demographics where  appropriate). | | Tables with each participant’s demographic information listed line-by-line. | The ‘Study Population’ section addresses the participant data collected and all reported demographic data has been aggregated to protect participant anonymity. Age groups (65-70 years, 71-75 years, etc.), rather than individual ages, were collected and median deprivation scores for the group were reported without posting individual residential locations. Participant demographics were not reported with individual quotes as this was not appropriate or relevant to the study analysis. |
| Detail ethical approval and ethical code/principles followed, participant informed consent, etc. | Ethical discussion usually includes institutional ethical approval (if needed), but may include wider principles; providing research materials (participant information, consent form, etc.) in supplementary materials may be useful to support reflexive  openness. | | Compromising participant anonymity by the details provided. | The Method section homes an Ethical Considerations sub-section. The institutional ethical approval is documented alongside the discussion of key issues such as consent and debriefing. Research materials, such as the recruitment poster and the interview topic guide, are available upon request. |
| **Dataset generation** NB: We prefer the term generation over collection to capture the active role of the researcher and that data don’t pre-exist research as data, but become data through research practices. | | | | This study refers to data generation within its own heading in the Method section, acknowledging that our approach does not presume that data pre-exists but is generated by conducting the study itself. |
| Provide some rationale for method(s) for data generation/data item sources chosen. | Discuss why the method(s) of data generation/data source was a good fit with the research question, participant group, guiding theory, etc. If multiple data sources are used, any rationale for combination should be conceptually appropriate (e.g.,  crystallisation). | | Triangulation as a rationale for different data sources (realist). | The Data Generation section of the Method describes the methods used and why they were appropriate (e.g. offering interview slots seven days a week to avoid shift pattern bias). Justification for the use of interviews as a method has been provided in reflection of the research aim and objectives and the need for rich individualised data. |
| Describe development and/or characteristics of data generation tool(s). | Include tool(s) in supplementary materials when possible; discuss piloting if used, and any changes following piloting, or during  data generation. | | Using an existing tool with the aim of replicating existing  “findings”, or developing and describing a tool in a way that is intended to facilitate future  replication (positivist). | The topic guide can be found in the Supplementary Materials, which formed the basis of the interviews conducted. Existing research studies were used to inform the development of the tool; this was not completed to replicate existing findings but instead to identify topics that spurred open conversations in the interviews. |
| Include details such as modality and/or setting of data generation, time frame, and other pertinent procedural information. | Relevant information includes: the mode of a data generation tool (e.g., video call focus groups; chat-based interviews); the context of data generation (location; timeframe) – where this  doesn’t compromise participant anonymity; and mode of recording interactive  data generation. | | Standardisation as a gold standard (realist); justifying an aimed for standardisation in data generation tools as a means to facilitate the  “reliability” or “accuracy” of the research; treating a lack of standardisation in data generation method, modality or setting as a problem, a  potential source of “bias”. | The Method contains modality details including the process around audio recording and transcribing interviews. The location and timeframe of the data generation are also noted. A detailed data management plan was developed in the process of gaining institution ethical approval. This plan included detailed consideration of participant anonymity and data storage, as highlighted in the manuscript. |
| Describe who conducted any interactive data generation (which author or research role), and how. | Can include what, if anything, the researcher disclosed about their personal or professional positioning or motivation; what skills and experience they brought; note  researcher’s relationship with  participants prior to, during and after the research. | | Seeking standardisation (e.g., through the training of researchers) in interactive data collection; treating non- standardisation as a threat to “reliability” or “accuracy”. | The researcher did not know the participants prior to the study and the researcher’s role and motivation were communicated to participants at every stage of the research. This was not a blind study and the institution association was transparent. The relationship between the researcher and the participants was only during the period of data generation and follow-up. |
| Describe the size/scope of  dataset and dataset items. | Such as the range and average  length for interviews/focus groups; range and average word length for textual data  items. | | Equating data quantity with  data quality. | The Method section details the interview recordings, including the number, mean duration and range duration. |
| Describe, and if relevant explain, any preparation of data for analysis. | Such as method of transcription of audio/video data (a transcription key can go in supplementary materials); changes and “corrections” – such as why typographical errors in written data were corrected; system for removing any identifying information; use of pseudonyms and/or data  codes. | | Describing transcription as “verbatim” or “orthographic” with no further details; using edited or “cleaned up” data without acknowledgement of this; participant validation of the “accuracy” of transcripts (realist). | The Method section details the transcription process and use of associated software. All transcripts were checked for accuracy, typographical errors corrected and anonymised prior to analysis. As part of the Data Management Plan, the process of removing identifiable information, such as names, with generic ‘XXXX’ codes is detailed. The transcription was described as ‘verbatim’ as an overall approach, but with supporting details around how this was achieved. |
| **Data analysis** | | | |  |
| Provide some rationale for use of RTA, and, where relevant, for combining RTA with other approaches and procedures. | Any combining of RTA with other method/ologies or procedures should be warranted, rather than based on a misunderstanding of RTA, and conceptually coherent  (unless clearly justified). | | Citing generic characteristics of RTA (e.g., accessible, flexible) without explaining how they were relevant to the study; using a codebook without acknowledging this is not part  of RTA and justifying its use. | Reflexive thematic analysis was used in isolation as an analysis technique that underpinned an essentialist/realism approach. The rationale for using reflexive thematic analysis is detailed in the Method section; it allowed the researcher to develop themes pertaining to the factors that influence, underpin or contextualise older people’s behavioural motivations in diet and antimicrobial use. The approach was taken with extensive use of Braun and Clarke’s publications from 2006 to 2024 to encourage a methodologically coherent process. A codebook was not used and the generation of themes followed a realist informed approach that is described in detail. |
| Describe specific orientation to RTA. | Locate RTA on dimensions of inductive<>deductive and semantic<>latent. | | A generic discussion of TA (or even RTA), not specifically situated in relation to the study  or approach. | Braun and Clarke’s practical approach to developing themes was followed throughout the data generation process. The Method section describes how developing the codes and data analysis places the reflexive thematic analysis as a variant of the inductive approach using abduction and retroduction.  Thematic maps were developed in NVivo to consider how provisional themes related to each other and the overall research question before themes were refined, defined and conclusively named. |
| Discuss how the researcher(s) engaged with the analytic process. | Provide a specific and situated account of the analysis process; use supplementary materials to provide a fuller account of the analytic  process. | | Offering a generic description of the six phases of RTA in lieu of an account of analytic process. | The Data Analysis sub-section provides a detailed account of the analysis process. The standard 6-step approach from Braun and Clarke was followed, but this was not described in a generic way and rather was reported as the process pertaining to this individual study and its benefits to achieving the research aim and objectives. |
| Where more than one person is involved, describe who analysed the data (author or research role). | Role(s) or involvement throughout the process should be discussed; where coding was collaborative, what this involved and how differences in coding and theme development were tackled,  should be included. | | Use of inter-coder agreement measures, consensus coding approach (positivist). | A single researcher analysed the data and associated collaboration was not present; however, code ideas were discussed with all authors. The concepts of an inter-code agreement and additional coders were rejected as not part of a reflexive thematic analysis process. |
| Use language to describe the process and products of RTA that is coherent with the values and assumptions of RTA. | Language should convey the active role of the researcher(s) in “generating”, “crafting”, “constructing”, “creating”, “producing” or “developing” themes; language around themes should evokes them as products of a researcher-data process. | | Passive language of discovery, such as “emerging”, “found”, “identified”, “discovered” – these evoke themes as “diamonds scattered in the sand” (p. 740)10; unexplained use of language and concepts from other approaches, such as emergent or superordinate  themes (IPA), or line-by-line and/or open coding and constant comparison  (grounded theory). | The language used throughout the research is one of active development and care was taken to avoid thinking or reporting on the data in alternative formats. |
| **The Analysis**  NB: We prefer the heading Analysis over Findings/Results. Findings implies the researcher “found”, “discovered” or “identified” pre-existing themes. Results is strongly associated with the outputs of statistical analysis. | | | | The heading ‘Results’ has been used in line with the journal’s conventions, but care has been taken throughout the section to report themes in an active way and not as findings that were ready to be found or discovered. |
| ***Reporting the data analysis*** | | | |  |
| Provide an overview of themes or thematic structure. | Overviews can include a list, map or table of themes to  preview the analysis. | | An unclear thematic structure, including unexplained headings  in the Analysis. | A table of themes (Table 2) is included at the start of the Results section. |
| Ensure theme conceptualisation is appropriate to RTA, and any divergences are justified and  explained. | In RTA, themes report shared meaning, united around a central organising concept that differs for each theme. | | Topic summaries; data generation questions reported as “themes”. | The theme conceptualisation was based on various organising concepts that were not directly tied to questions or topic summaries. Instead, they adhered to the realist approach, creating causal mechanisms derived from events and experiences identified during the analysis. |
| Name themes appropriately. | Use theme names that capture the “essence” or  “story” of each themes; brief  data quotations can be used. | | (One-word) theme names that only identify a topic, and offer no story (evoking topic  summaries). | A modified approach was used, where the realist approach encouraged the research to generate themes as causal mechanisms, articulated in longer, explanatory statements. These were combined with shorter theme names, drawn from quotations in the data, to capture the essence of the participants' shared experiences. |
| Report themes in sufficient depth and detail. | As RTA is an interpretative method, themes should be multifaceted, and contain both data and analytic narrative; if useful, additional data extracts may be included  in supplementary materials.7 | | Thin, one dimensional themes, effectively conflating codes and themes; large number of themes relative to the length of the manuscript. | Five core themes were developed, each representing a distinct dimension of the events and experiences, aiming to reflect the complexity of the process from various perspectives. Data extracts were included to give voice to the participants and illustrate different elements of the themes in detail. |
| Use subtheme judiciously. | Themes are the main analytic purpose, and should be multifaceted; only use subthemes where doing so highlights an important facet or aspect of the central  concept of a theme. | | Fragmenting the analysis through overuse of subthemes, and an overly elaborated/“bitty” thematic structure. | Table 2 showcases the key events and experiences identified in the analysis that were used to create the themes. These events and experiences serve as subtheme headings, identifying 2-3 different components that highlight the various facets within each theme, accompanied by supporting data extracts. |
| Ensure the analytic narrative explains the meaning and significance of the data. | For RTA, each theme needs an analytic narrative that outlines its meaning and importance in relation to the topic, research question and dataset; the reader needs to be told about why/how data excerpts matter and “evidence” the theme; the Analysis section also needs to convey the overall story of the analysis. | | Frequency counts as a justification for themes presented; simple paraphrasing of data as “analytic narrative”; treating data meaning as self- evident (data are assumed to speak for themselves); “arguing” with the data (treating the data as something to [dis]agree with, rather than to interpret and make sense  of). | Each theme is detailed in the Results section, accompanied by an analytic narrative and linked to examples from the dataset. This approach ensures clarity for the reader regarding how each theme was developed and its connection to the data extracts. Numerical counts or frequency are not utilised as a justification for themes, in line with the reflexive thematic analysis approach. The Results section focuses solely on the analysis of the dataset without referencing or debating other research, which is addressed in the Discussion section. |
| Provide an appropriate balance of analytic narrative and data extracts – both data extracts and analytic narrative  matter. | The rich descriptive and/or interpretative story of the analysis needs to be woven around sufficient analytic extracts from across the  dataset. | | Presenting either a long string of data extracts with barely any analytic narrative, or only the researcher’s narrative summary of the theme, without any data  extracts to support it. | The analytic narrative is balanced with data extracts to let participants' voices come through and provide the rich description needed as part of the reflexive thematic analysis approach. These extracts are contextualised within the narrative to explain their role in supporting theme development. Additionally, data extracts are edited to avoid lengthy excerpts while preserving the conveyed sense and meaning. |
| Demonstrate coherence between analytic narrative and illustrative/evidentiary  data extracts. | Data extracts should convincingly and compellingly evidence the analytic claims. | | Mismatches between data extracts and analytic claims; not countering obvious  alternative readings of the data | The analytic narrative throughout the Results examines areas where data extracts converge and diverge, offering supportive examples of both. It also explores alternative explanations for why certain phenomena may occur. |
| Integrate existing research and theory into the analytic narrative. | In RTA, an interpretative analytic narrative is enriched by incorporating relevant existing research and theory into the reporting of themes, reflecting notions of contextualised meaning, and  contributing to an ongoing “conversation” about a topic. | | The positivist tradition of separating a description of analytic “Results” and their interpretation with reference to scholarship and theory in a “Discussion” section. | The Discussion section integrates research and theory into the analytic narrative. This approach diverges somewhat from the recommended reflexive thematic analysis methodology, as the Results section focuses solely on the dataset from this study without comparing it to other research. In this paper, the analysis is split over two headings: the Results centres on theme development specifically from the generated dataset, while the Discussion provides further analysis and situates it within the broader context of existing literature. Theme development acknowledges existing literature, as a reflexive thematic analysis researcher does not claim to be unaware of prior knowledge but uses it within epistemology to develop themes and causal explanations. This structure offers greater clarity for the reader, illustrating how the themes were developed from the data and what the data revealed. |
| **The Final Section – A General Discussion or “Conclusions”**  NB: We don’t have a preference for what a final section of an RTA report is called, and it depends on the context and the focus and purpose of the study – the heading Conclusion may evoke a certainty that isn’t appropriate; Implications may be useful; Final Considerations or Reflections may work, as might General Discussion. | | | | The term ‘Discussion’ was used as a heading to reflect journal conventions; however, an analytic approach to the research and write-up was taken to avoid suggestions to a degree of certainty in the data. |
| *Quality, evaluation and conclusions* | | | |  |
| Draw analytic conclusions across themes. | Orient to the “so what” of the overall analysis – the “point” of the story told; this might include discussion of implications for practice and  “actionable” outcomes. | | Repetitive theme-by-theme integration of the analysis with existing literature; no overall conclusions drawn; no overall analytic story. | The Discussion revisits the original aim and objectives of the research, including addressing the implications of the findings in terms of AMR management strategies, and provides a discussion of specific recommendations for public-related ARM management. This section aims to serve as a "so what" segment, justifying the research and its relevance within the broader literature. |
| Discuss implications or directions for future research. | Any suggestions for future research should stem from the analysis and be evidence- based (e.g., provide grounds for other groups potentially having different experiences  or views) rather than generic. | | Generic recommendations for other research, such as with a different “population”. | Suggestions for further research have been offered and these have been grounded in the codes and themes developed. For example, further research into the generational perspectives of AMR. |
| Use and report quality practices coherent with RTA. | Ensure evaluation of research quality deploys conceptually coherent notions, such as: member reflections; crystallisation; others serving as a critical friend/sounding board to enhance insight;  reflexive journaling. | | Incoherent quality measures such as: member checking/participant validation; triangulation (realist); the use of theme agreement/consensus among researchers or corroboration of themes by  another researcher (positivist). | This table has been utilised to reflect on the quality of the research and its reporting, ensuring a rigorous methodological approach. Input from all authors acting as critical friends was incorporated to discuss ideas throughout the research process, from inception to completion. Positivist concepts such as member checking, triangulation, and inter-rater reliability were not employed in this research. |
| Evaluate the research from a Big Q standpoint. | Such evaluation might include considering how the specifics of the study may have shaped the research produced (for example, the characteristics and context of the participant group/dataset; the methods and modalities  for generating the data); situatedness should not be  treated as a limitation. | | Evaluations and descriptions of limitations that orient to quantitative or positivist norms, such as reference to lack of generalisability – positioned as a limitation, and equated only with statistical generalisability – or a “small” (by implication non-ideal) and  “unrepresentative” “sample”. | The limitations of the study are outlined in a specific ‘Study Strengths and Limitations’ sub-section. Volunteer and recall bias are highlighted as potential weaknesses in the method. This research used an older population; the more abundant recruitment of individuals from the younger end of the age threshold has been considered, as has the influence of recruiting from community centres. |
| Include reflections on research process and practices, including researcher reflexivity. | Some consideration of the researcher(s)’s role in shaping the research and the knowledge generated is an important quality marker. | | Reference to researcher bias/influence (positivist). | A first-person account of the research process has not been included as it does not align with the third-person preference of journal writing. However, the paper acknowledged the researcher’s role in knowledge generation and how this has situated the research in the bigger picture. |
